# 2024 HRS perspective on advancing workflows for CIED remote monitoring

**DOI:** 10.1016/j.hroo.2024.09.012

**Published:** 2024-09-27

**Authors:** David J. Slotwiner, Gerald A. Serwer, James D. Allred, Deepak Bhakta, Richard Clark, Julien Durand, Martha G. Ferrara, Jason Hale, Chris Irving, Andy Iverson, Maobing Jin, Jens B. Johansen, Matthew Kalscheur, Dennis Krisjnen, Robert Lerman, Neal Lippman, G. Stuart Mendenhall, Ryan Michael, Steven Nichols, Ratika Parkash, Noemi Ray, Craig Reister, Nicholas T. Skipitaris, Harry Solomon, Paul R. Steiner, Marko Tietz, Elaine Y. Wan, Manish Wadhwa

**Affiliations:** 1 NewYork-Presbyterian Queens, Flushing, New York; 2 Division of Cardiology, Weill Cornell Medical College, New York, New York; 3 Congenital Heart Center, C.S. Mott Children’s Hospital, University of Michigan Health, Ann Arbor, Michigan; 4 CV Remote Solutions, Greensboro, North Carolina; 5 Indiana University School of Medicine, Indianapolis, Indiana; 6 MicroPort CRM USA, Arlington, Tennessee; 7 Implicity, Paris, France; 8 White Plains Hospital, White Plains, New York; 9 PaceMate, Alexandria, Louisiana; 10 Murj, Santa Cruz, California; 11 Medtronic Inc, Minneapolis, Minnesota; 12 Abbott, San Jose, California; 13 Department of Cardiology, Odense University Hospital, Odense, Denmark; 14 Division of Cardiovascular Medicine, Department of Medicine, University of Wisconsin School of Medicine and Public Health, Madison, Wisconsin; 15 Fysicon, Oss, North Brabant, The Netherlands; 16 Peerbridge Health, Los Angeles, California; 17 Arrhythmia Consultants of Connecticut, LLC, Hartford, Connecticut; 18 Scripps Memorial Hospital La Jolla, La Jolla, California; 19 Vector Remote Monitoring, Bend, Oregon; 20 GE HealthCare, Chicago, Illinois; 21 Dalhousie University, Halifax, Nova Scotia, Canada; 22 Boston Scientific, Arden Hills, Minnesota; 23 Department of Cardiology, Lenox Hill Hospital, Northwell Health, New York, New York; 24 Dartmouth Hitchcock Medical Center, Lebanon, New Hampshire; 25 Biotronik, Portland, Oregon; 26 Division of Cardiology, Department of Medicine, Vagelos College of Physicians and Surgeons, Columbia University, New York, New York; 27 Philips, San Diego, California

**Keywords:** Interoperability, Standards, Cardiac implantable electronic device, Implantable cardioverter-defibrillator, Fast Healthcare Interoperability Resources, Health Level 7

## Abstract

Cardiac implantable electronic devices (CIEDs) generate substantial data, often stored in image or PDF formats. Remote monitoring, now an integral component of patient care, places considerable administrative burdens on clinicians and staff, in large part due to the challenge of integrating these data seamlessly into electronic health records. Since 2006, the Heart Rhythm Society, in collaboration with the CIED industry, has led an initiative to establish a unified standard nomenclature. This effort has harmonized terminology, aligning diverse terms with single terms approved by the Institute of Electrical and Electronics Engineers. With this foundational work complete, attention now turns to developing technical standards for interoperability, which would enable the smooth communication of CIED data between information technology systems used in clinical practice. In this article, by leveraging Health Level 7 Fast Healthcare Interoperability Resources, we present a road map for the technical committee to guide this endeavor. We identify critical data exchange points between remote transceivers, electronic health records, and third-party platforms commonly used for CIED patient data management. Our objective is to establish bidirectional communication among these resources, ensuring the accuracy, timeliness, and accessibility of clinical data for clinicians. We also anticipate substantial benefits for both clinical research and administrative efficiency through the implementation of this interoperability framework.

## Section 1: Introduction

Managing patients with cardiac implantable electronic devices (CIEDs) requires collecting, analyzing, and effectively handling large amounts of data produced by these devices. Unfortunately, these data are often isolated in proprietary formats that are not interoperable, leading to the need for multiple incompatible medical information management systems to manage patients’ CIED data.^1^ To address these challenges, clinical practices have been compelled to adopt time-consuming data management workflows, often involving the allocation of human resources for repetitive and mundane tasks.

**Figure 1:**
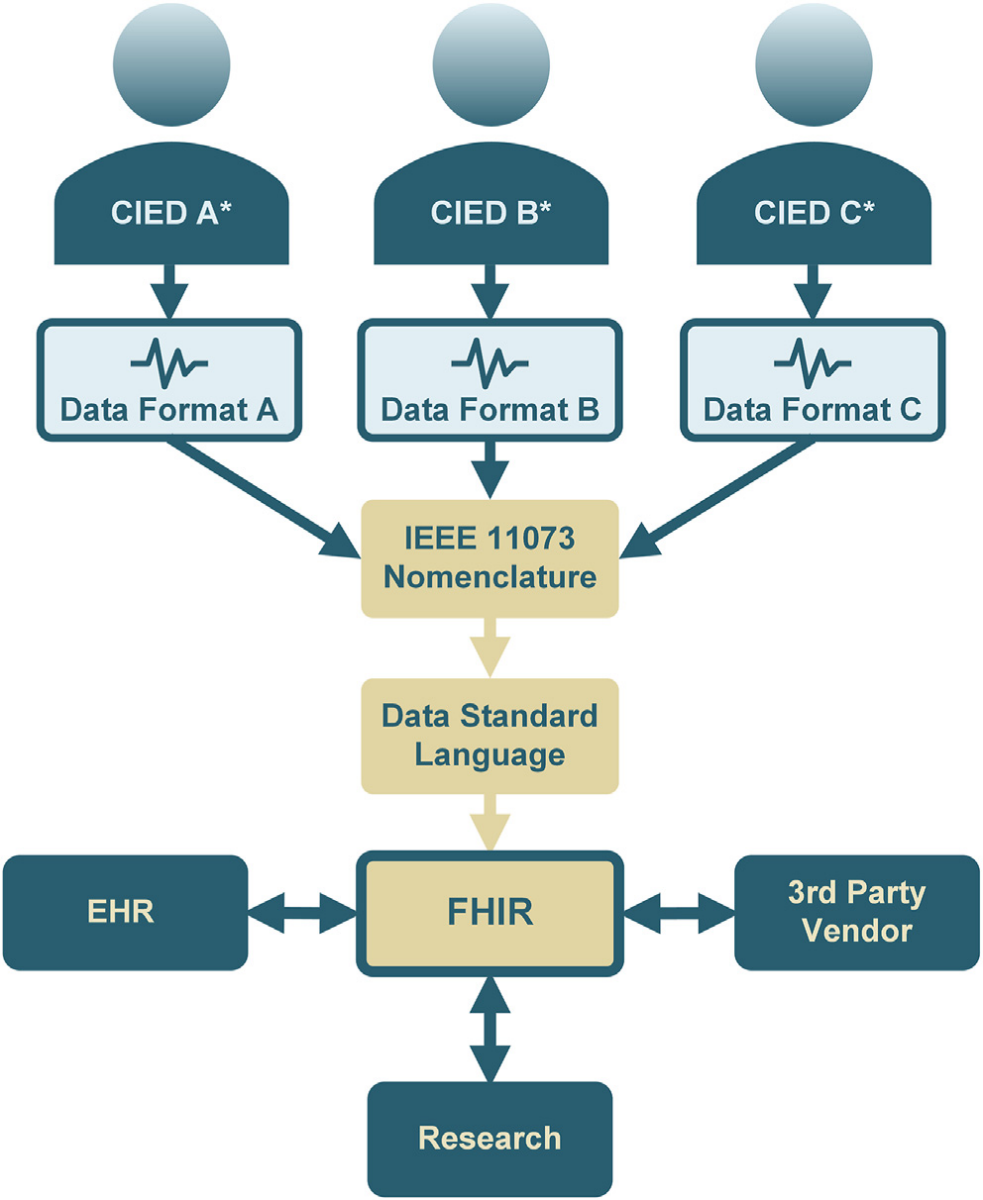
Cardiac implantable electronic device (CIED) manufacturers A, B, and C∗ each use their own unique nomenclature and data formats to describe and record CIED functions and parameters. The Institute of Electrical and Electronics Engineers (IEEE) 11073 nomenclature, developed by the Heart Rhythm Society in collaboration with CIED manufacturers, standardizes these proprietary formats into a universally compatible system, endorsed by the international standards organization IEEE. Additionally, FHIR (Fast Healthcare Interoperability Resources) is a standard created by the Health Level Seven International for electronic health care information exchange. It specifies how health care information can be shared across different computer systems, irrespective of the storage method, thereby facilitating secure and efficient data access and sharing among health care providers. EHR = electronic health record.

Section 2 of this document presents examples of various clinical scenarios related to CIED data management, where workflow inefficiencies are frequently encountered. For each scenario, opportunities to enhance efficiency and establish a more reliable CIED data workflow are discussed (Figure 1). The consistent theme across these scenarios is the identification of solutions that fundamentally depend on (1) the semantic standardization of CIED data elements and (2) the implementation of interoperability standards among the different workflow components needed for optimal patient care.

Existing interoperability standards, such as International Organization for Standardization (ISO) and Institute of Electrical and Electronics Engineers (IEEE) standard 11073.10103-2012 and Integrating the Healthcare Enterprise (IHE) standard Implantable Device Cardiac Observation (IDCO) v1.0, which can improve CIED workflows, are being revised with thoughtful updates and enhancements.^2^ Section 3 of this document summarizes these revisions and provides a brief historical overview of the development of these crucial standards endorsed by the Heart Rhythm Society (HRS).

Standardizing data elements and ensuring interoperability between the necessary components for patient care and CIED data management is expected to enhance both reliability and workflow efficiency. This will free up resources, allowing more effective patient care.

## Section 2: Use cases

**Figure 2:**
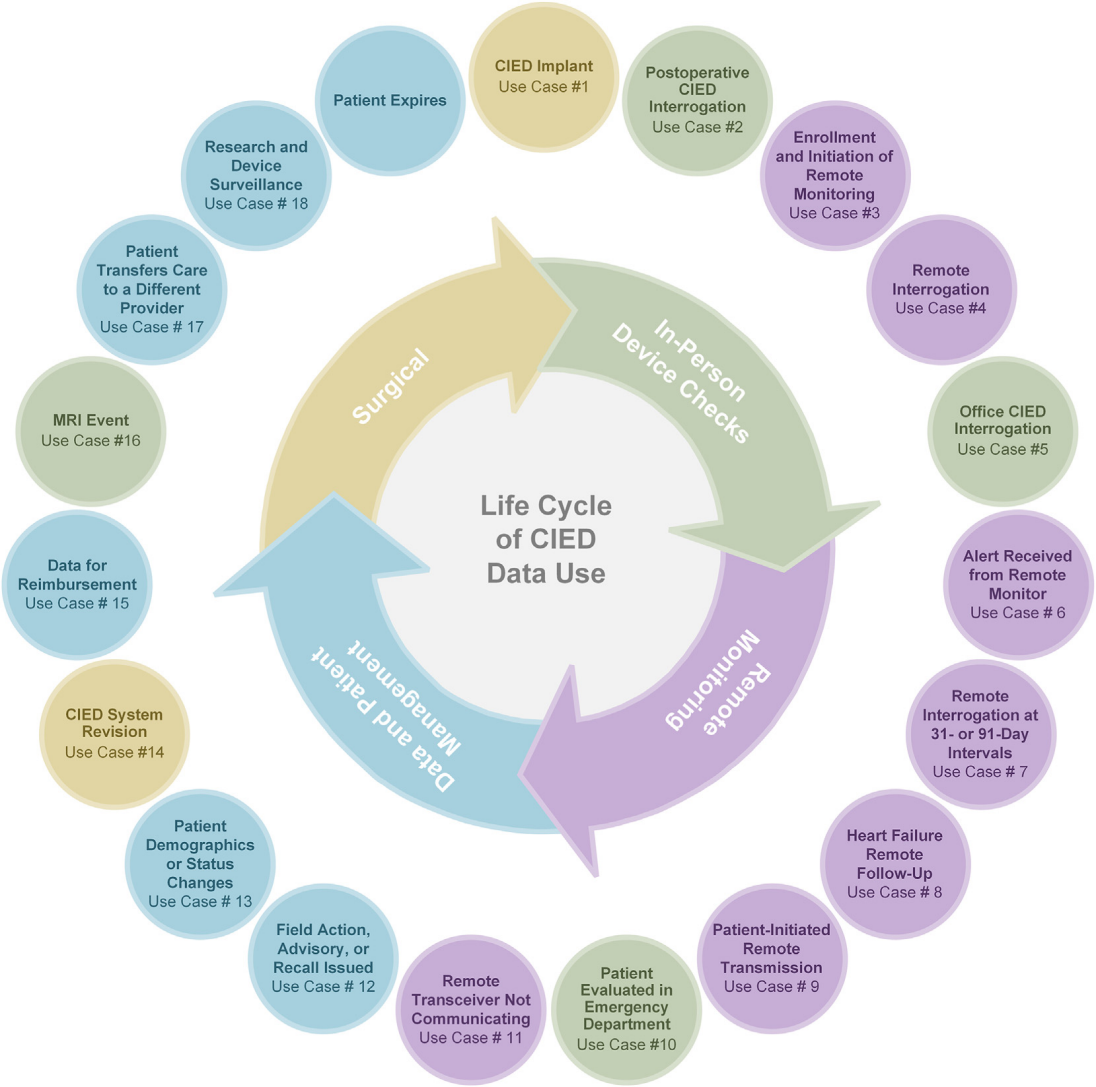
Life cycle of cardiac implantable electronic device (CIED) data use. The instances during a patient’s care when data related to the patient, the CIED, or both are typically generated and should be communicated to all information technology systems used for managing the care of the patient. MRI = magnetic resonance imaging.

The following section outlines the instances during a patient’s care when data related to the patient, the CIED, or both are typically generated (Figure 2). We also discuss the potential benefits that could be realized if standardized data elements were used and if bidirectional communication was established between information technology (IT) systems.

Components of data management include the following:

• CIED
• Remote transceiver
• CIED programmer
• CIED vendor remote monitoring portal
• Third-party CIED data management system (DMS)
• Electronic health record (EHR)

### Use case 1: CIED implant

Before the procedure, data that validate the need for the implant are gathered and, if suitable, the process of shared decision making is documented. Details about the procedure, such as device hardware, collected data, and device programming, are obtained and noted. Additionally, information regarding the patient’s demographics and the implanting physician is collected. At the end of the procedure, an operative note is created, the implant is registered with the manufacturer, and a patient identification card is generated.

#### Opportunity

Use of standardized data elements enables seamless communication of clinical data between the EHR and the third-party DMS. This scenario emphasizes the need to transfer data collected by the manufacturer’s CIED programmer to a third-party CIED DMS, an EHR, or both. It is crucial to record and store the model, serial number, and unique device identifier of each component of the implanted CIED system in both the EHR and the DMS. Using standardized data elements to represent measured data and device settings, coupled with interoperability among IT components, minimizes the risk of errors and ensures efficient data communication.

### Use case 2: Postoperative CIED interrogation

Before being discharged, a patient’s newly implanted CIED system is interrogated. The data obtained from the CIED programmer are then transferred to both the EHR and the DMS, establishing a baseline for addressing future CIED management issues.

#### Opportunity

Use of standardized data elements and bidirectional communication facilitates the seamless transfer of clinical data between the EHR and the DMS, thereby preventing errors in data transfer. This ensures that health care professionals can make well-informed clinical decisions without consulting multiple IT systems while also guaranteeing that the recorded data remain current and accurate. It is important to highlight that CIED settings may need to be adjusted later on, as settings for the acute stage may differ from settings for the subacute stage of inflammation at the distal lead sites of myocardial fixation. After the lead implant site has matured, the EHR and/or DMS can be set up to send reminders for optimizing CIED settings.

### Use case 3: Enrollment and initiation of remote monitoring

Enrollment and initiation of remote monitoring requires several steps, including patient education, obtaining patient consent, configuring the CIED and the remote transceiver, and establishing a schedule for remote transmissions.

#### Opportunity

Bidirectional communication between the transceiver, remote monitoring vendor server, EHR, and DMS will allow providers to manage the care of a patient in either the EHR or the DMS and will ensure that the data are updated simultaneously across IT systems.

### Use case 4: Remote interrogation

The process of remote interrogation ensures that the remote monitoring functionality is correctly linked to both the specific hardware and the individual patient. It also sets up a continuous schedule for future follow-ups. The clinic reviews the collected data and compares them with the data gathered during the implantation procedure and/or the postoperative interrogation. This data review can be conducted within the DMS or EHR.

#### Opportunity

It is crucial to keep the data in the DMS and EHR accurate and current. This ensures that future interrogations and decision making are based on precise information, regardless of whether a practice or practitioner reviews the data in the DMS or EHR. Using standardized data elements and bidirectional communication facilitates this process, ensuring reliable and consistent data maintenance.

### Use case 5: Office CIED interrogation

Patients present to the office for routine evaluation of their CIED. A report is automatically prepared, and after review and signature by the responsible clinician, it is forwarded to the EHR. Additionally, a report specific to each patient is generated and sent to them.

#### Opportunity

This example highlights the critical role of previously obtained data in the evaluation and management of patients with CIEDs. Accurate and easily retrievable information aids clinicians in efficiently caring for these patients and in preventing catastrophic outcomes in vulnerable individuals. Proper recording, along with automated transmission and storage of baseline and follow-up measurements, enables clinicians to compare real-time data with previous values. This is crucial in diagnosing system-related issues and identifying progressive abnormalities that may signal impending system failure. Conditions such as lead dislodgment, electrode malfunction, and premature battery depletion are examples where these comparisons are especially valuable.

### Use case 6: Alert received from remote monitor

An alert is generated after routine remote interrogation when an abnormal condition or a significant change is detected. This includes conditions such as dramatically increased electrode impedance, elevated pacing thresholds, battery depletion, or the detection of a significant arrhythmia. The DMS and EHR are promptly notified, and they generate a report. The clinic is then required to acknowledge receipt of the alert through the CIED vendor remote monitoring portal.

#### Opportunity

This scenario underscores a critical challenge clinics face in managing these alerts: the need for timely data availability and seamless communication between all IT systems used in patient care. To achieve this, standardized data elements and interoperability are essential.

### Use case 7: Remote interrogation at 31- or 91-day intervals

This use case is similar to use case 6, with the key difference being its scheduled nature. In this scenario, data communication occurs routinely at either 31-day intervals or 91-day intervals. The data must then be transferred to the DMS and EHR, followed by the creation of a report.

#### Opportunity

As with use case 6, ensuring that data are available and current across all IT systems managing a patient’s device is crucial. The routine nature of this use case emphasizes the importance of maintaining up-to-date and accurate data for effective patient device management.

### Use case 8: Heart failure remote follow-up

Use case 8 is similar to use cases 6 and 7; however, in this example, heart failure sensor data are communicated.

#### Opportunity

As for use cases 6 and 7, it is crucial that data are available and up to date in all IT systems used to manage a patient’s heart failure to prevent avoidable consequences including hospitalization.

### Use case 9: Patient-initiated remote transmission

Use case 9 is similar to use cases 6, 7, and 8. Patient-initiated remote transmission allows the patient to notify the device clinic of their concerns. These transmissions may be the result of arrhythmic symptoms, implantable cardioverter-defibrillator shock delivery, or a patient’s detection of audible or vibratory alerts from a CIED; patients usually receive an expedited response.

#### Opportunity

It is important that data are available and up to date in all IT systems used to manage a patient’s device and to address acute patient needs and symptoms.

### Use case 10: Patient evaluated in emergency department

A patient is evaluated in the emergency department, and an interrogation of their CIED is performed. These data may be important for providers to access and therefore must be available in the EHR.

#### Opportunity

It is important that data are available and up to date in all IT systems used to manage a patient’s device.

### Use case 11: Remote transceiver not communicating

This scenario involves interruptions in communication, either between the remote transceiver and the vendor’s remote monitoring server or between the CIED and the remote transceiver.

#### Opportunity

This situation is one of the most critical and time-consuming challenges encountered in remote monitoring practices and poses significant patient safety concerns. It exemplifies how interoperability between IT systems can offer substantial improvements. Practices lack the resources to continuously monitor each vendor’s remote monitoring portal for noncommunicating transceiver alerts. Ideally, these notifications would be directly communicated to both the DMS and the EHR. After acknowledgment of the notification in the DMS or EHR, confirmation would be sent back to the vendor’s remote monitoring portal. This process ensures that all IT systems have accurate and current data. It also allows efficient allocation of staff resources to reestablish the communication of the remote monitoring transceiver.

### Use case 12: Field action advisory, or recall issued

When a field action advisory is issued for a CIED component due to an observed higher-than-expected failure rate, it warrants immediate attention. Patients with the affected component might be asymptomatic until failure occurs, potentially leading to serious symptoms or events. The manufacturer identifies the source of the potentially faulty component, which is typically isolated to a subset of products within a specific range of serial numbers. Clinicians receive management recommendations for affected patients, including intensified monitoring and, if necessary, component replacement. This scenario, which is somewhat common, raises concerns for both patients and clinicians. It requires a delicate balance of mitigating the risk of serious complications in affected patients while avoiding unnecessary interventions. Accurate recording of a CIED patient’s component information—including model types and serial numbers—is crucial to rapidly identify those at potential risk of system failure, enabling the prescription of an appropriate management plan.

#### Opportunity

In such situations, rapid identification of at-risk patients is crucial to prevent serious outcomes. Automated transfer of component details to the EHR at the time of implantation facilitates the identification of affected patients. While recording system information is the initial step, integrating these data within the EHR offers the added benefit of combining system data with clinical patient details. This integration helps to segment the at-risk patient population and identify those at particularly high risk of serious complications who may require more aggressive management strategies. Conversely, available and expanded remote monitoring capabilities could be applied to patients at relatively lower risk, for whom conservative management strategies are more appropriate. Additionally, implementing a 2-way interface for adjusting routine report scheduling in the EHR would enable the easy intensification of remote monitoring for patients as needed.

### Use case 13: Patient demographics or status changes

When there are changes to a patient’s demographic information or in the event of a patient’s death, the EHR is updated to reflect these changes. Efficiently transmitting this updated information from the EHR to the DMS and the vendor’s remote monitoring portal will enhance the accuracy and timeliness of patient data documentation, including vital status. This improvement is crucial for enhancing future clinician-patient communications and avoiding complications arising from a lack of awareness about the patient’s current condition. Similarly, if the DMS is informed of a change in the patient’s situation, it can relay this update to both the EHR and the vendor’s remote monitoring portal.

#### Opportunity

Establishing 2-way communication between the DMS, EHR, and the vendor’s remote monitoring portal is essential. It not only ensures the accuracy of patient information but also increases the efficiency of clinical staff.

### Use case 14: CIED system revision

When hardware replacement is necessary, the existing hardware information can be retrieved and referenced in the procedure note. Additionally, details of the newly implanted and abandoned equipment can be documented. The functional characteristics of the new reporting system will facilitate the direct transfer of all information from the testing equipment to the procedure note. This updated information is then communicated across all IT systems, including the DMS, EHR, and remote monitoring portal.

#### Opportunity

The process of direct automated data transfer improves the accuracy of information related to the system components. It significantly reduces the need for manual data entry, thereby minimizing the potential for human error and further conserving resources and time.

### Use case 15: Data for reimbursement

When a patient’s CIED transmits, either for an alert or for a routine report, the transmission must be put into a schedule context to determine billing eligibility. If the report is used for billing, a patient’s future scheduling may need to be verified or modified so that future routine reports meet current procedural terminology guidelines. To alleviate manual management of a patient’s schedule on a device manufacturer’s web portal, one strategy is to schedule a patient for a set number of routine transmissions within a 31- or 91-day cycle and “accumulate” the monitoring days to meet Medicare guidelines. While this works well to alleviate precise ad hoc scheduling, it increases the transmission frequency from a device, which in turn increases clinical workload and may affect battery longevity.

#### Opportunity

A 2-way interface for manipulation of a patient’s routine report scheduling would allow programmatic optimization for both transmission volume and workload and battery longevity.

### Use case 16: Magnetic resonance imaging event

A patient undergoes magnetic resonance imaging, and the CIED is temporarily reprogrammed for the procedure as recommended by the manufacturer and the clinician managing the CIED.

#### Opportunity

The CIED logs the programming changes and the magnetic resonance imaging event and communicates the information at the time of the next remote monitoring transmission. This information is shared between the vendor server, EHR, and DMS to keep up-to-date and accurate information in all systems.

### Use case 17: Patient transfers care to a different provider

A patient elects to transfer care to a different provider or group of providers/health care system. Access to all historical and present data pertaining to the patient’s CIED should be made available to the new providers.

#### Opportunity

Presently, with components of data related to the management of a CIED located in different IT systems, transferring all pertinent data to a new provider is difficult. By ensuring that all data are stored in an interoperable EHR, transferring the data will be streamlined.

### Use case 18: Research and device surveillance

CIED systems from all manufacturers are potentially susceptible to malfunction. Generator and lead malfunctions have occurred due to latent design flaws or the discovery of adverse operation long after implantation. Currently, the detection and reporting of system malfunctions are primarily voluntary and often done through the U.S. Food and Drug Administration Manufacturer and User Facility Device Experience (MAUDE) database (https://www.accessdata.fda.gov/scripts/cdrh/cfdocs/cfMAUDE/search.CFM). Manufacturer-based postmarketing surveillance plays a role in identifying component failures. However, the effectiveness of this surveillance largely depends on the manufacturers’ ability to observe and report these failures. Having structured, granular data accessible in the EHR, and pooling this information, will significantly enhance CIED system surveillance in clinical research. This approach will allow earlier identification of CIED component malfunctions.

#### Opportunity

This approach could lead to a significant shift in post–market approval device surveillance. Instead of relying on small vendor-based cohort studies, it would enable population-wide surveillance. This means all patients with a specific CIED and/or lead could be monitored for signs of device or lead malfunction. Such malfunctions might become apparent through increased health care resource use. This could include more frequent device interrogations or CIED system revisions, an uptick in inappropriate shocks from implantable cardioverter-defibrillators, or other unusual patterns in health care resource use.

## Section 3: Evolution of HRS work on CIED data elements and interoperability

### Timeline

Work on interoperability began in 2006 when the HRS joined forces with the American College of Cardiology and IHE.^3^ This collaboration aimed to bring clinicians and industry engineers together to address challenges in clinical medicine through technological solutions. Among the many initiatives, the one that garnered significant interest and support focused on managing the extensive data from CIEDs, especially data gathered via remote monitoring. This initiative, known as the IDCO profile, aimed to enable the secure and standardized retrieval and storage of CIED data, regardless of the device manufacturer, and an overview of the process has been previously published.^4^ The timeline for the initiative is as follows:

• 2006: Working group started: Biotronik, Guidant, Medtronic, St. Jude Medical
• 2007: White paper published: The Role of the Heart Rhythm Society in Integrating the Healthcare Enterprise
• January 2010: First Connectathon
• June 17, 2010: First ballot for the ICDCO v1.0 nomenclature initiated
• August 27, 2012: IEEE nomenclature standard published (ICDCO v1.0)
• February 13, 2014: ISO nomenclature standard published (ICDCO v1.0)
• December 5, 2015: IEEE Project Authorization Request for the IDCO 1.1 nomenclature approved (workgroup for IDCO 1.1 started)
• December 2, 2022: First ballot for the IDCO 1.1 nomenclature initiated

### IDCO 1.1

The technical working group, established in 2006, operated under the IEEE Project Authorization Request, currently P11073-10103. This group continues to conduct weekly meetings to define new parameters, basing their work on priorities identified in surveys of HRS clinicians. Initially, the group drafts a detailed proposal for each parameter, including its representation in the nomenclature. This proposal is then presented to the HRS Interoperability Workgroup. After a discussion, the group considers any suggested updates or recommendations, revising the proposal as needed. This process is repeated and the updated proposal is presented to the HRS Interoperability Workgroup until they are satisfied with the parameter details for implementation. Once a sufficient number of parameters are defined, the technical working group compiles these into technical documents. These documents are then submitted to the relevant governing body, either IEEE or IHE, for approval before publication.

#### Minimal data set according to device type

The IEEE nomenclature standard, approved on August 27, 2012 (ICDCO v1.0), initially faced a challenge: it did not ensure that a minimum data set was included with each device encounter.^2^ Consequently, vendors began implementing only limited data elements from the nomenclature. This led to a misconception among users that the nomenclature was incomplete and lacked the detailed information clinicians needed for device management. The actual issue was the vendors’ failure to report many of the IEEE nomenclature fields. To address this, between 2018 and 2020, the HRS Interoperability Workgroup and the technical working group developed recommendations for mandatory and optional data elements in the implanted cardiac device data sets, tailored to each device type (eg, implantable pulse generator, implantable cardioverter-defibrillator, and implantable loop recorder). The standards technical experts believed that these specifications should be included in the IHE profile rather than the nomenclature. Currently, the recommendation to incorporate these into the IDCO profile is under review and awaiting approval.

#### New in the IEEE/ISO nomenclature for IDCO 1.1 (expanded capabilities and notifications)

The IEEE/ISO nomenclature, which provides a comprehensive overview data set, marks a good beginning in standardizing device information. However, it falls short in standardizing some important details. Since 2015, the technical group in collaboration with HRS has been dedicated to adding more standardized terms. Presented below is the list of the updated nomenclature, which was submitted in 2022 and approved in 2023.

○ Advisory-related information for device and leads
○ Universal device identifier for device and leads
○ Battery longevity time frame (defines the ability to represent battery status until the relative remaining time is reached)
○ Battery relative remaining time timestamp
○ Multisite pacing status and LV-LV delay setting
○ Multisite pacing location, amplitude, and pulse width settings
○ Blanking and refractory periods
○ AF suppression algorithm status and name
○ Right ventricular pace avoidance status and name
○ Ventricular rate during ATAF statistics
○ Ventricular rate during mode switch statistics
○ Antitachycardia pacing (ATP) and shock statistics
  ▪ Shocks successful – recent
  ▪ Shocks successful total
  ▪ ATP successful recent
  ▪ ATP successful total
  ▪ Atrial ATP delivered recent
  ▪ Atrial ATP delivered total
  ▪ Atrial ATP successful recent
  ▪ Atrial ATP successful total
  ▪ Ventricular ATP delivered recent
  ▪ Ventricular ATP delivered total
  ▪ Ventricular ATP successful recent
  ▪ Ventricular ATP successful total
○ Intervals during episodes (atrial and ventricular)
○ ATP and shocks delivered during the episode (number and energy)
○ Episode in progress
○ Notifications

### Gaps in workflow

Although they represent a major step forward, ICDCO v1.0 and IDCO 1.1 leave some challenges to be addressed. Three challenges are detailed below.

1. Devices evolve faster than the standard
  • Device manufacturers are continually adding new sensors or enhancing existing ones, thereby increasing the data available for patient care in various medical fields, including electrophysiology and cardiology. For example, Biotronik has integrated body temperature sensors and Boston Scientific has incorporated respiratory rate sensors. Consequently, some CIED manufacturers are proactively expanding the IDCO by adding these new data elements while others adhere strictly to the existing standard. It is essential for standards to include guidelines on how new information can be incorporated and eventually standardized. Looking ahead, we need to develop paradigms that allow quicker updates of the standard and frameworks, enabling vendors to incorporate innovative proprietary features without diverging from the standard.
2. Data trends are not available in IDCO implementation
  • IDCO makes it possible to report measurements as single values measured on a given date and time or averages computed over a period.
  • Since remote monitoring typically relies on a 30-day or even 90-day period, communicating a single value or an average over the whole period can be of little clinical relevance, as it will not reflect fast or temporary changes that may require attention.
  • This design makes it possible to report trends by reporting a series of single values.
  • Unfortunately, this approach to trends is not formally endorsed by IDCO. Therefore, CIED manufacturer implementation varies greatly (and is often limited to averages).
3. Nonstandardized information cannot be represented in a standardized message
  • As stated previously, some CIED manufacturers extend IDCO by proactively adding data elements. This has been done in various ways, including mixing standard and nonstandard data elements. To improve interoperability, a framework could be developed for how standards can be extended, and this type of proprietary information included should be defined.

## Section 4: Fast Healthcare Interoperability Resources accelerator

### Emergence of Health Level 7’s Fast Healthcare Interoperability Resources accelerators CodeX and Cardiovascular data eXchange

To develop interoperable standards for the use cases described in this article, the workgroup suggests using the Fast Healthcare Interoperability Resources (FHIR, pronounced “fire”) implementation of the Health Level 7 (HL7) standard (Figure 1). There is substantial evidence of lasting support for FHIR standards from both implementers and regulatory bodies. These development efforts will coordinate with IHE/HL7 workgroups and HL7 FHIR accelerators as appropriate.

HL7, established in 1987, is an international not-for-profit organization that aims to create a comprehensive, standardized American National Standards Institute–affirmed framework. This framework is designed for the integration, exchange, sharing, management, and retrieval of electronic health information. The 7 in HL7 refers to the top layer of the Open Systems Interconnection networking model, denoting the application protocol. This implies that HL7 does not rely on the lower layers and can use any protocol, whether wireless, hardwired, Transmission Control Protocol/Internet Protocol, or other technologies. HL7 formats the data exchanged between high-level applications, offering flexibility independent of computer architecture or network specifics. HL7 version 2, developed in 1987, is a common mechanism for exchanging administrative and clinical information, including the IDCO profile. HL7 also manages additional specifications like FHIR.

FHIR began in 2012 as an open health care interoperability standard. It uses common web technologies for structuring and exchanging information, contrasting with the proprietary technology required for HL7 version 2 messages. FHIR adopts a modern approach, specifying the use of the lower presentation layer or structures using JavaScript Object Notation or Extensible Markup Language. It specifies exchanges through a modern application programming interface, typically using standard secure Internet technologies for communication.

Web technologies facilitate easier data sharing, including that with patient mobile devices. In the United States, certified health technologies must support FHIR and FHIR-based applications. The FHIR standard is developed and balloted within HL7 working groups.

IHE and HL7 share common goals and have a formalized working relationship to promote standards and interoperability. Several IHE profiles based on HL7 messaging are being refactored to FHIR, and new FHIR native IHE profiles are under development.

To support specific medical communities and groups in adopting FHIR, “FHIR accelerators” have been established. These focus on key use cases, starting with underlying standards for personal health records. Accelerators maintain open participation and may receive logistical and organizational support from HL7 as needed.

The oncology community, for example, developed the mCODE (Minimal Common Oncology Data Elements) FHIR Implementation Guide, addressing specific patient care and clinical research use cases through the CodeX FHIR accelerator. Building on CodeX’s success, a new cardiovascular domain–focused initiative has emerged. The aim of CodeX is to be the FHIR accelerator for clinical domain–specific interoperability.

Goals of the project include the following:

1. To create valuable real-world evidence that is interoperable, which relies on normalized EHRs consistently defining disease phenotypes, treatments, and outcomes. Currently, real-world cardiology data often depend on clinical registry reporting, which lacks concordance in basic concepts such as birth sex and smoking history. For instance, an analysis of 21 clinical registries found 6 primary variations in the value set for birth sex.^5^ Common data models such as Observational Medical Outcomes Partnership and the National Patient-Centered Clinical Research Network are efforts to standardize observational data but often miss key cardiology concepts. The reliability of *International Classification of Diseases, Tenth Revision* codes in observational studies is also questionable, particularly for specific conditions.^6^ Patient-reported outcomes are limited in clinical trials and practice.^7^ The CardX (Cardiovascular data eXchange) domain of the CodeX HL7 FHIR Accelerator project aims to drive the cardiology community toward interoperability for robust real-world evidence insights.
2. To identify major cardiac data beyond laboratories and procedures that should flow from the EHR with the patient, such as New York Heart Association functional classification. Opportunities lie in (a) consistent indications for procedures, including defining patient disease state/phenotype and (b) patient-reported symptoms at the time of procedures and in follow-up, potentially including adverse events/complications.

## Section 5: World forum for CIED follow-up

In 2022, the European Heart Rhythm Association (a branch of the European Society of Cardiology) and the HRS collaborated with the Asia Pacific Heart Rhythm Society and the Latin American Heart Rhythm Society. This partnership aimed to address international concerns about the challenges of managing the extensive data generated by CIEDs. The goal was to pressure manufacturers and third-party DMS vendors to collaborate further in developing and implementing data standards and interoperability solutions. The first meeting, held in Barcelona in 2022 alongside the European Society of Cardiology Congress, saw attendance from leaders of each organization. At the end of the session, there was unanimous agreement on the need for an international approach to this high-priority issue. A second meeting took place in New Orleans, coinciding with the HRS 2023 annual meeting. There, all stakeholders signed a statement of common goals. This statement expressed the commitment to implementing the existing IEEE nomenclature and continuing collaboration to create a truly interoperable CIED data stream. This effort aims to benefit patients worldwide and establish a foundation for the continuous growth of the CIED field.

## Summary and next steps

Significant progress has been made in developing a single, internationally accepted nomenclature for CIED data. This achievement has gained recognition and support from international electrophysiology societies, CIED manufacturers, and third-party vendors. All these stakeholders are committed to implementing the existing IEEE nomenclature to support semantic interoperability. Furthermore, they are dedicated to ongoing collaboration to develop technical solutions. These solutions aim to enhance the safety, reliability, and efficacy of care for patients with CIEDs. The next step involves developing bidirectional communication among vendor CIED servers, third-party DMS, and EHRs. This will ensure the availability of consistent, accurate data across all IT systems managing CIED patient data. It will also ensure the full implementation of use case scenarios outlined in this article. We believe that the CardX domain of the CodeX HL7 FHIR Accelerator is ideally positioned to facilitate this collaborative effort.
