# Supplementary material for: 2024 HRS perspective on advancing workflows for CIED remote monitoring
Source: Heart Rhythm O2. 2024 Sep 27;5(12):845–53. doi: 10.1016/j.hroo.2024.09.012 (PMC11721723; doi:10.1016/j.hroo.2024.09.012)
Supplement: Appendix 1 [file mmc1.pdf]

Appendix 1 Author disclosures

Name	Employment	Honoraria/ Speaking/ Consulting	Speakers' bureau	Research*	Fellowship support*	Ownership/ Partnership/ Principal/Majority stockholder	Stock or stock options	Intellectual property/ Royalties	Other
David J. Slotwiner, MD, FHRS (Chair)	NewYork-Presbyterian Queens, New York, New York, and Division of Cardiology, Weill Cornell Medical College, New York, New York	None	None	None	None	None	None	None	None
Gerald A. Serwer, MD, FHRS	Congenital Heart Center, C.S. Mott Children's Hospital, University of Michigan Health, Ann Arbor, Michigan	None	None	None	None	None	None	None	None
Deepak Bhakta, MD, FHRS, CCDS	Indiana University School of Medicine, Indianapolis, Indiana	None	None	1; Medtronic, Inc.	None	None	None	None	None
Martha G. Ferrara, DNP, FNP, FHRS, CCDS	White Plains Hospital, White Plains New York	None	0; Boston Scientific Corp.	None	None	None	None	None	None
Matthew Kalscheur, MD	Division of Cardiovascular Medicine, Department of Medicine, University of Wisconsin School of Medicine and Public Health, Madison, Wisconsin	None	None	None	None	None	None	None	None

Neal Lippman, MD, FHRS	Arrhythmia Consultants of Connecticut, LLC, Hartford, Connecticut	1; Boston Scientific 3; Abbott	None	None	None	None	None	None	None
G. Stuart Mendenhall, MD	Scripps Memorial Hospital La Jolla, La Jolla, California	1; Medtronic, Inc.	None	None	None	None	1 ; Grektek, LLC	None	None
Ratika Parkash, MD, MS, FHRS	Dalhousie University, Halifax, Canada	0; Medtronic, Inc., 1; Servier	None	4; Novartis, 5; Abbot, 7; Medtronic, Inc.	None	None	None	None	None
Nicholas T. Skipitaris, MBA, MD, FHRS	Department of Cardiology, Lenox Hill Hospital, Northwell Health, New York City, New York	1; Medtronic, Inc., Biotronik GE Healthcare	None	None	None	None	None	None	None
Paul R. Steiner, MD, FHRS	Dartmouth Hitchcock Medical Center, Lebanon, New Hampshire	None	None	None	None	None	None	None	None
Elaine Wan, MD, FHRS	Division of Cardiology, Department of Medicine, Vagelos College of Physicians and Surgeons, Columbia University, New York, New York	1; Sanofi, Medtronic, Abbott, Boston Scientific, Cardiologs	None	7; National Institutes for Health	None	None	None	None	None

Number value: **0** = \$0; **1** = ≤ \$10,000; **2** = > \$10,000 to ≤ \$25,000; **3** = > \$25,000 to ≤ \$50,000; **4** = > \$50,000 to ≤ \$100,000; **5** = > \$100,000.

*Research and fellowship support are classed as programmatic support. Sources of programmatic support are disclosed but are not regarded as relevant relationships with industry for writing group members.
